# Temperature Dependence for Survival, Development, and Reproduction of the Cactus Cochineal *Dactylopius opuntiae* (Cockerell)

**DOI:** 10.3390/insects13050426

**Published:** 2022-04-30

**Authors:** Mohamed El Aalaoui, Mohamed Sbaghi

**Affiliations:** 1 Regional Center of Agronomic Research of Settat, Tertiary Road 1406, At 5 Km from Settat, Settat 26400, Morocco; 2 Plant Protection Department, National Institute of Agricultural Research, Ennasr, Rabat 10000, Morocco; msbaghi@yahoo.fr

**Keywords:** *Dactylopius opuntiae*, biological parameters, reproduction parameters, thermal requirements, population trend index

## Abstract

**Simple Summary:**

Cactus is an important drought-tolerant crop that has very various beneficial uses. In Morocco, cactus is a perfectly appropriate crop for land rehabilitation in the arid and semi-arid regions. With little investment, it can produce important resources for human and animal nutrition and generate adequate revenues for farmers. Unfortunately, the sustainability of this extremely resilient ecosystem has become seriously threatened by the appearance of *Dactylopius opuntiae* (Hemiptera: Dactylopiidae). This cochineal which is specific to cacti, was introduced in Morocco in 2014. The aim of this study was to evaluate the effect of temperature on life cycle parameters of *D. opuntiae* at 20, 23, 26, 32, and 40 ± 1 °C, 65 ± 5% RH, and a photoperiod of 12L:8D. Temperatures ranging from 26 °C to 32 °C were appropriate for the survival, development, and reproduction of the scale insect. Parthenogenesis in females was not observed during our study. This study also showed that *D. opuntiae* females required a higher thermal constant (769.23 D°) than males (357.14 D°) to grow to the mature adult stage.

**Abstract:**

The effect of temperature on *Dactylopius opuntiae* (Cockerell) life cycle parameters was evaluated at 20, 23, 26, 32, and 40 ± 1 °C, 65 ± 5% RH, and a photoperiod of 12 L:8 D. Temperatures ranging from 26 °C to 32 °C were suitable for survival, development, and reproduction of *D. opuntiae*. The total developmental time of females ranged from 94.23 d (20 °C) to 43.55 d (40 °C). The average development time of males from egg to death ranged from 26.97 days at 32 °C to 50.75 days at 20 °C. The probability that a newly laid egg would survive to the adult stage was highest at 26 °C and 32 °C (44–60%). The parthenogenesis in females was not observed during our study. The longest oviposition period was observed when the cochineal was reared at 32 °C (17.97 days), and the highest fecundity was observed at 32 °C (355.29 egg/female). The highest proportion of females (0.80) was observed at 40 °C. According to the age-stage-two-sex life table, the highest value of the intrinsic rate of natural increase (r_m_) was recorded at 32 °C. The lower developmental thresholds for the total pre-adult female and male and adult female and male stages, were 10.15, 12.21, 10.54, and 21.04 °C, respectively. *Dactylopius opuntiae* females needed a higher thermal constant (769.23 D°) than males (357.14 D°) to achieve their development and reach the mature adult stage. These findings will be useful for the development of an integrated pest management strategy for *D. opuntiae*.

## 1. Introduction

The false carmine scale *Dactylopius opuntiae* (Cockerell, 1986) (Hemiptera: Dactylopiidae) has been proven to be the most damaging insect pest of cactus species and is responsible for massive economic losses worldwide [1,2]. Originally from Mexico, this invasive scale has spread to many zones around the world, among them the Mediterranean basin, including Israel, the Gulf region [3], and Morocco [4]. According to several researchers, the significant climate changes that the Mediterranean region has undergone in recent years, notably the increase in temperatures, have played an important role in increasing desertification; they predict that the region will become drier and hotter [5]. These changes will undoubtedly affect all parts of the agro-ecosystem [6] and will favor the survival and development of harmful insects and indirectly affect the trophic relationships (hosts and natural enemies) [7]. It is therefore expected that a scale pest such as *D. opuntiae* constitutes a serious threat to cactus cultivation in countries that have not yet been attacked (the scale pest has been detected more recently in Algeria and Tunisia).

*Dactylopius opuntiae* attack both prickly pear cactus fruits and cladodes [8]. The cochineal affects approximately 16 cactus species in different geographical regions in the world. It causes economic losses of millions of dollars annually, either through production loss or pest control costs [5,9,10]. *Dactylopius opuntiae* establishes and spreads more easily than many other cochineal species due to many reasons, including the waxy coating on their back surface that protects them from insecticides, high fecundity, and the propensity to spread rapidly by natural means such as wind, water, rain, birds, humans, farm animals, etc [11].

Because of the small size of the first instar nymph, which is the potential stage of infestation, it is extremely difficult to detect the cochineal in the early stage of infestation [12]. The cochineal tends to form colonies of variable size on fruits and cladodes [13] and tends to prefer areas that are less exposed to light [14], with some preferences depending on the host species. For example, Mann [14] observed that in Australia, *D. opuntiae* primarily infests basal stems of *Opuntia stricta* (Haw.) Haw., 1812 and main stems of *Opuntia tomentosa* Salm-Dyck, 1822. Nymphs and female adults of *D. opuntiae* feed directly on the plant, causing chlorotic yellowish areas and necrosis on fruits and cladodes, then it dries out [15] in about a year, although the stems may survive for a few more months [2]. Furthermore, the trophic activity of the scale pest can weaken the plant, and consequently favor attack by pathogens that can cause their death [16]. *Dactylopius opuntiae* has been intentionally introduced in certain countries for the biological control of invasive cactus weeds [5]. 

The catastrophic damage caused by *D. opuntiae* in several countries in the world requires an integrated pest management (IPM) approach that also takes into account the importance of protecting biodiversity that could be affected by the introduction of alien organisms [17]. This approach includes several methods based on genetic, biological (natural enemies), chemical, mechanical, physical, and other methods [17,18,19] that can be combined in various ways to obtain the best results in the control of this harmful pest.

Several factors can hinder the survival and development of pests [14]; among them, the temperature can directly influence the reproduction and abundance of scale pests [20,21,22]. The temperature has an important effect on the life cycle parameters of a wide range of insects; thus, each insect has an optimal temperature range for development and reproduction [23], and their survival and development are significantly affected when the temperature is above or below this range [24]. The lower development threshold (LDT) and the sum of effective temperatures (SET) have often been used to explain how insect establishment, development, and reproduction are dependent on temperature [25,26,27]. It has been assumed that a nonlinear relationship between insect development rate and temperature is obtained at higher temperatures [28], and in the favorable temperature range, this relationship is almost linear [29]. These variations in the relationships help in predicting the LDT, the SET, the dynamics of insect populations, and also in determining the best method of control, the timing of the release of natural enemies, or application of insecticides to achieve successful pest control.

Very superficial and incomplete studies have been generated for many species of Dactylopiidae viz., *Dactylopius ceylonicus* (Green 1896) [30], and *Dactylopius austrinus* (De Lotto, 1974) [31], and yet no studies on the effects of temperature on survival, development, and reproduction of *D. opuntiae* have been reported. In this study, the life table parameters of *D. opuntiae*, including its survival, development, and reproduction on *Opuntia ficus-indica* (L.) Miller., 1768 in environmental growth chambers at five constant temperatures are reported.

## 2. Materials and Methods

### 2.1. Establishment of D. opuntiae Colony

*Dactylopius opuntiae* used in the cultures were from individuals collected from cactus plantations in the locality of Zemamra in the Casablanca-Settat region (33°15’ N, 8°30’ W), Morocco. Were placed on *Opuntia ficus-indica* cladodes of 1-year-old and about 3–4 kg inside the cages (80-80-80 cm) comprised of a wooden frame covered with a mesh fabric to allow ventilation under controlled conditions at 26 ± 2 °C, 60 ± 10% relative humidity and an L:D regime of 12:12 h. To increase the number, and to follow the age of the insects, the “cut cladode technique” of Aldama-Aguilera and LlanderalCázares [32] was adopted. 

### 2.2. Biological Parameters

The experiment regarding the development of *D. opuntiae* was conducted under 5 constant temperatures: 20, 23, 26, 32, and 40 °C (±1 °C). Before starting the life table at each temperature, *Opuntia ficus-indica* cladodes, susceptible to *D. opuntiae* (one year old and of approximately 3.5 Kg), were harvested in March 2021 in the experimental field station (32°15′ to 33°15′ N, 7°55′ to 9°15′ W) at INRA Settat, Morocco plantation and have been allowed to air dry for 10 days at a temperature range from 17 to 28 °C then were planted in normal polarity in plastic pots (33 cm diameter by 12 cm height), filled with a mixture of fine sand (2/3) and peat (1/3), and allowed to grow until the plants reached the stage of two to three cladodes in a greenhouse (11 m × 7 m) at a temperature of 28 °C/17 °C (day/night), and then the plants were transferred and placed in an environmental growth chamber set at test temperatures, relative humidity of 65%, and a photoperiod of 12:8 (L:D) h. The greenhouse temperature data were calculated from the thermograms, based on 6 measurements made with intervals of 2 h. The night temperature was determined from the 3 lowest daily values [33]. Plants were irrigated as needed. Twenty one-day-old first instar nymphs of *D. opuntiae* (obtained from eggs of adults of the two-trophic rearing system) were carefully transferred with a fine camel hair brush (No. 000, CAMLIN, Tanis Inc, 3660 Kettle Ct E, Delafield, Wisconsin 53018, US) to the plants (20 nymphs per plant considered as a replicate) and replaced at the defined temperature in the growth chamber. Ten replicates were maintained at all temperatures. Plants were examined twice daily (09:30 h and 16:00 h) to determine the success of the insect’s installation and accumulated development times and survival rates of the current stage. The nymphs became less mobile after the crawlers settled on a suitable feeding site on the cladode. However, as body length and shape appear to be well correlated with developmental stages [12,34], the following morphological characteristics obtained in a previous study by El Aalaoui et al. [12] were used to separate nymph instars: newly hatched first-instar crawlers were small (0.83–1.11 mm length, 0.47–0.53 mm width) and bright red in color. The sex of each nymph was determined at the second instar nymph stage, after which the developmental cycle and morphology differed for males and females. *Dactylopius opuntiae* male has 5 biological stages: egg, nymph (1st,2nd,3rd,4th, and 5th), pre-pupa, pupa, and adult. The last 3 nymphal stages (3rd,4th, and 5th nymph) develop inside cocoons [12]. To avoid ambiguity, the combined development duration of male nymph (3rd, 4rd, and 5th instars) was considered as the cocoon stage for analysis. At the end of the cocoon stage, a red, white-winged male emerged. *D. opuntiae* females have 3 biological stages–egg, nymph (2 instars), and adult [12]. The first wax secretions produced by second-instar female crawlers initially appeared as white dust that gradually elongated into white coils [12,34]. The wax increased in quantity with age, forming a thick, white, cottony cushion that eventually concealed the developing female insect [12,34]. The second molt took place underneath this covering, and the white exuviate was displaced to the edge of the covering. *Dactylopius opuntiae* females are 4.67 mm long and 2.67 mm and subglobular in shape. The mean mass of *D. opuntiae* females is 26.33 mg [12]. 

Observations on nymph development to determine the duration of pre-adult life stages, cumulative development time from crawler to adult emergence for both males and females, sex ratio (proportion of females), total adult longevity for both sexes, biological and survival data of each stage, and survival to adult emergence were recorded. To ensure the reproducibility of results, all experiments were independently repeated twice over time.

### 2.3. Reproduction Parameters

In a separate experiment, *D. opuntiae* fecundity and fertility were studied at 20, 23, 26, 32, and 40 °C (±1 °C). *Opuntia ficus-indica* cladodes, susceptible to *D. opuntiae* (one year old and of approximately 3.5 Kg), were collected in a greenhouse plantation at the experimental field station at INRA Settat, Morocco, dried and planted, and allowed to grow until the plants reached the stage of two to three cladodes in the greenhouse at a temperature of 28 °C/17 °C (day/night) as described above (Biological parameters studies section). The plants were transferred and placed in an environmental growth chamber set at test temperatures, relative humidity of 65%, and a photoperiod of 12:8 (L:D) h, then each plant was infested by 20 *D. opuntiae* first instar nymphs that had been produced at the same temperature, and we let the scale pests develop. At the gravid female’s stage (before a few days of starting oviposition), only females were kept in each plant, and cocoons (males) were removed using a needle [35]. The design and replication of the study were the same as in the ‘‘Biological parameters studies section’’; each plant (20 insects) was considered as a replicate, and ten replicates were maintained at all temperatures. Each female was observed daily until death, which allowed us to record their longevity, the length of the adult pre-oviposition period (APOP) (the period from young female to first oviposition or mature female), the total pre-oviposition period (TPOP) (the period from egg to mature female), the oviposition and post-oviposition periods. Adult female longevity was the sum of the adult pre-oviposition, oviposition, and post-oviposition periods. Fecundity (number of eggs laid) and fertility (number of eggs with embryos) of females were also recorded. To determine the hatchability %, eggs and crawlers were counted daily and discarded. Hatchability (%) was calculated using the following equation reported by Abbas et al. [36]:

Hatchability=All neonates/All eggs

The same experiment was conducted to test whether *D. opuntiae* reproduces parthenogenetically. Twenty crawlers were transferred to each plant. All males were removed as soon as they pupated, and only females were allowed to develop. Female size changes were assessed visually in all tested temperatures, as well as the females were examined daily for oviposition. To ensure the reproducibility of results, all experiments were independently repeated twice over time.

### 2.4. The Cochineal Thermal Requirements

To determine the thermal requirements of *D. opuntiae*, the lower developmental threshold (LDT) and the sum of effective temperatures (SET) were estimated using the developmental times of the different stages of the cochineal at each constant temperature (20, 23, 26, 32, and 40 °C). Honék and Kocourek [29] reported that in the range of temperatures favorable for insect development, the relationship between the inverse of development time (development rate) and temperature is nearly linear. Given this information, the thermal summation model [20,37] was used to estimate the linear relationship between temperature and the rate of development of *D. opuntiae*. The following linear relationship equation was used:

(1) 1/DT=aT+b

where 1/DT is the development rate (DT is development time) of the developmental stage, which is proportional to the temperature (T), and a (slope) and b (intercept) are the regression parameters. The LDT corresponds to temperature (T) when there is no development, that is when 1/DT = 0 [38]. The number degree-day (SET) required for development was calculating using SET = 1/a, where a is the slope of regression of 1/DT on temperature [37]. 

### 2.5. Life Table Parameters

Data on biological and reproductive parameters, the survival rate of different instars, sex ratio (proportion of females), and adult fecundity listed in Table 1, Table 2 and Table 3 were used to construct a life table for *D. opuntiae* at five constant temperatures according to Morris [39] as cited by Prasad et al. [25] by fixing the initial standard number of first instar nymphs at 200 for all temperatures. The expected fecundity of the next generation (G2) and the population trend index (I), which allow estimation of the population growth rate of the next generation relative to the initial number, were calculated [25,40].

Data on biological and reproduction parameters and adult longevity obtained at different constant temperatures (20 °C, 23 °C, 26 °C, 32 °C, and 40 °C) were estimated according to the age-stage two-sex life table [41,42] by the computer program TWOSEX-MSChart [43] and analyzed by one-way ANOVA followed by Tukey’s LSD test at α = 0.05 using SPSS software [44]. For differences between the duration of female and male stages on each temperature, Fisher’s LSD test was adopted for comparisons between significant treatment effects when they occurred, using STATISTICA software (ver. 6). Regression was conducted with XLSTAT (XLSTAT 2017) to determine the linear relationship between the development rate (1/DT) and temperature (T) and to estimate the regression parameters (a and b).

The age-stage, two-sex life table method was adopted to estimate life history data and population growth parameters of *D. opuntiae* on the five constant temperatures [41,42]. The cochineal life history data including: age-stage-specific survival rate (s_xj_) (where x = age, j = stage), age-specific survival rate (l_x_), age-stage-specific fecundity (f_xj_), age-specific fertility (m_x_), and population growth parameters including: R_0_, the net reproductive rate; r, intrinsic growth rate; λ, finite growth rate; T, mean generation time, and DT, doubling time. The net reproduction rate (R_0_), which represents the total number of offspring that an individual female can produce during its lifespan, was estimated as follows:

$$R=\sum_{x=0}^{\infty }\mathrm{Ixmx}$$

The intrinsic growth rate is approximated by the iterative bisection method from the Euler–Lotka formula with age indexed from 0 [45]:

$$\sum_{X=0}^{\infty }e^{-r\left(x+1\right)}\mathrm{Ixmx}=1$$

The mean generation time (the average time between two subsequent generations in the lineages of a population) is estimated as follows:

T=lnR0r

The finite rate of increase $\lambda ={\;e}^{r}\;$ and the doubling time DT=ln2/r were also calculated. In addition, the population sex ratio (proportion of females) and survival rate of *D. opuntiae* nymphs reared on the five constant temperatures were calculated using TWOSEX-MSChart. We used Sigma plot 14.5 to create graphs.

## 3. Results

### 3.1. Biological Parameters

Developmental periods of different stages of *D. opuntiae* were significantly affected by temperature (Table 1; *p* < 0.05). Within the range of temperatures tested, the average incubation period of eggs decreased significantly with increasing temperature; the average incubation period decreased from 23.82 h at 20 °C to 2.31 h at 40 °C (*F* = 13645.68, df = 4, *p* < 0.0001). After eclosion, the first-instar crawlers remained within the mesh of waxy filaments of the egg mass for a few minutes to a few hours before becoming mobile and leaving the threads to search for suitable settling and feeding sites in the cladodes. Most crawlers prefer to settle at the base of spines and away from light. The female total preadult period and longevity were significantly decreased as temperature increased from 20 to 40 °C (female preadult period F = 23,630.47, df = 4, *p* < 0.0001; female longevity F = 5480.47, df = 4, *p* < 0.0001). The male total preadult period decreased significantly with an increase in temperature in the range of 20–32 °C (F = 5715.83, df = 4, *p* < 0.0001). The shortest and longest adult male longevity were recorded at 40 °C (2.73 days) and 26 °C (7.85 days), respectively. The developmental periods in *D. opuntiae* females and males decreased significantly as temperature increased from 20 to 40 °C (female life cycle duration F = 14,637.76, df = 4, *p* < 0.0001; male life cycle duration F = 6829.33, df = 4, *p* < 0.0001). However, there was a significant increase in male life cycle duration between 32 °C (26.97 days) and 40 °C (31.91 days).

### 3.2. Survival Rate and Sex Ratio

The highest pre-adult survival was recorded at 32 °C (93%), followed by 26, 23, and 20 °C, respectively, and the significantly lower survival rate (28%) was recorded at 40 °C, but the highest sex ratio (proportion of females) (0.80) was observed at 40 °C (Table 2). 

**Figure 1:**
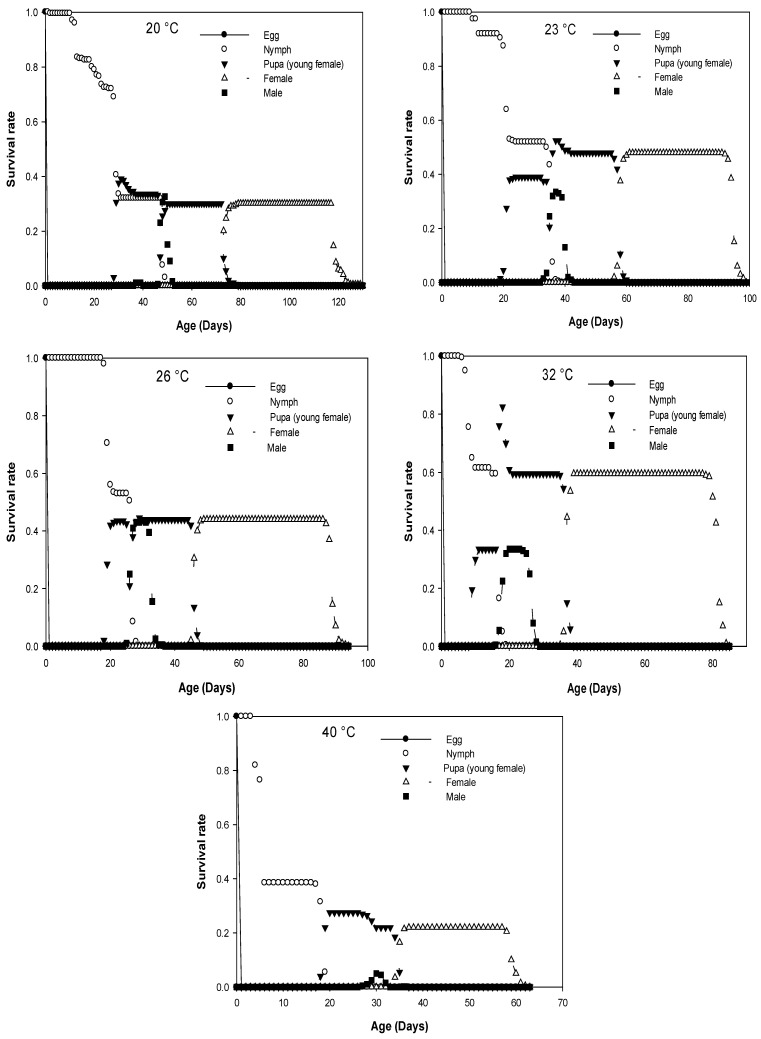
Age-stage specific survival rate (s_xj_) calculated for *D. opuntia* fed on *O. ficus-indica* at five different constant temperatures.

The age-stage-specific survival rate (s_xj_) indicates the probability that a newborn egg will survive to age x and stage j (Figure 1). Significant overlap between stages was observed for all temperatures tested due to the variable developmental rates among individuals. The probability that a newly laid egg would survive to the adult stage was as follows: 20 °C (0.30, 0.35); 23 °C (0.48, 0.34); 26 °C (0.44, 0.44); 32 °C (0.60, 0.34); 40 °C (0.22; 0.06) for females and males, respectively. The probability was the highest at 26 °C while it was the lowest at 40 °C for males. In the case of females, the percentage of survival from egg to adult development was the highest at 32 °C. There was no difference in this probability of survival between males and females at 26 °C.

### 3.3. Reproduction Parameters

**Figure 2:**
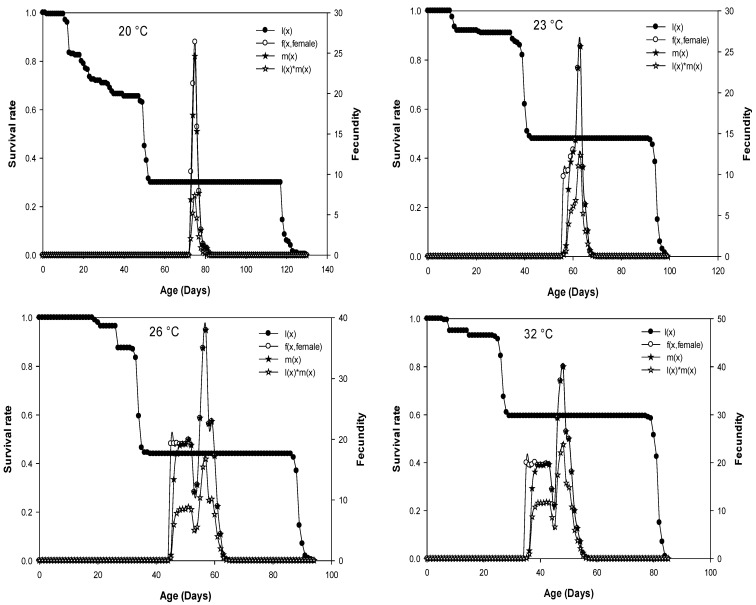

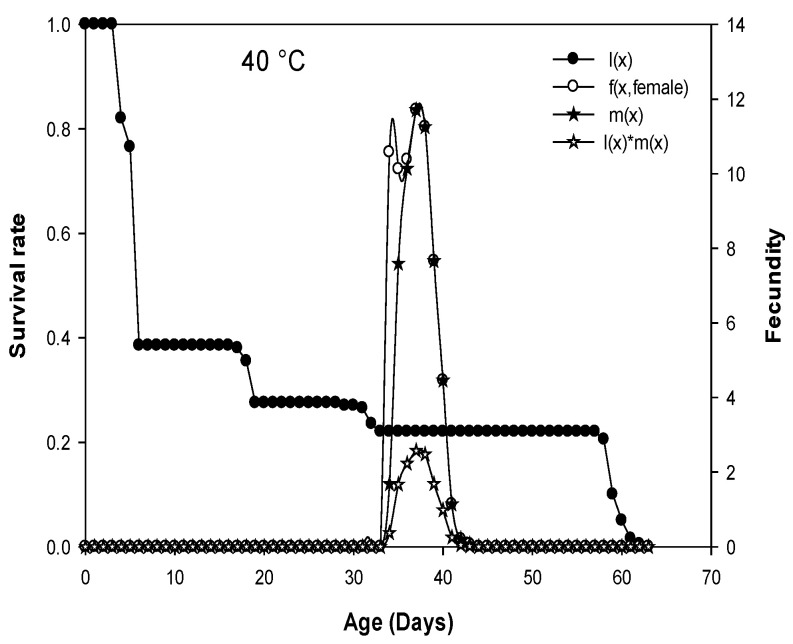
Age-specific survival rate (l_x_), age-stage fecundity of female (f_x_) (eggs/female), age-specific fertility (m_x_) (=born nymphs/female), and age-specific maternity (l_x_m_x_) of *D. opuntiae* fed on *O. ficus-indica* at five different constant temperatures.

The average number of eggs produced by a *D. opuntiae* individual of age x and stage j per day is plotted as age-stage fecundity (f_xj_) in Figure 2. Because only females produce offspring, there is only one curve f (x, female) that represents the females’ life-history stage. The age-specific survival rate curves (l_x_) describe the change in survival history of the whole cohort with age when all stages are represented, and age-specific fertility (m_x_) is the number of offspring born per female [20,21,22]. The age-specific survival curves (lx) that were presented in Figure 2 are temperature-dependent. The curves for age-stage specific fecundity (f_x_, female), age-specific fertility (m_x_), and age-specific maternity (l_x_m_x_) showed several peaks, with the highest peaks observed at 26 and 32 °C (Figure 2).

The presence of parthenogenesis in females was not observed in our study. No females in the absence of males produced eggs. The oviposition period was significantly affected by temperature (F = 12878.71, df = 4, *p* < 0.0001), its average varied from 4.72 to 17.97 days and was occasionally interrupted by 1 to 3 days without producing eggs. The longest oviposition period was observed when the cochineal was reared at 32 °C followed by 26 °C (16.82 days), and the shortest oviposition period was observed at 20 °C (4.72 days), which is nearly a quarter of that observed at 32 °C (Table 3). The total lifetime fecundity (number of eggs produced by each female during the oviposition period) was significantly affected by temperature and was significantly higher at 32 (355.29 egg/female) and 26 °C (325.02 egg/female) compared to the lower and the higher temperatures of 20 (78.37 egg/female) and 40 °C (55.89 egg/female) (F = 33552.84, df = 4, *p* < 0.0001) (Table 3). The egg hatching (%) was significantly higher at 32 °C (97.75%) and 26 °C (96.92%) and lower at 20 °C (61.55%) (F = 15694.91, df = 4, *p* < 0.0001) (Table 3).

### 3.4. Population Growth Parameters

The temperatures tested significantly affected the population parameters of *D. opuntiae* (Table 4). The intrinsic rate of increase (r_m_) of this scale pest increased significantly with increasing temperature from 20 to 32 °C (F = 172,759.29, df = 4, *p* < 0.0001). The greater value was achieved at 32 °C (0.119 d^−1^), while the lower level was obtained at 20 °C (0.041 d^−1^). The finite rate of increase (*λ*) showed a trend similar to r. The highest net reproductive rates (*R*_0_) were achieved at 32 °C (211.36 offspring per female), followed by 26 °C (143.00 offspring per female), while the lowest R_0_ was observed at 40 °C (12.30 offspring per individual female) (F = 5,420,551.49, df = 4, *p* < 0.0001).

Increasing temperature resulted in a shorter mean generation time (T) of *D. opuntiae*. This time was significantly higher at 20 °C (76.47 days) compared to the higher temperatures of 32 (44.43 days) and 40 °C (38.75 days) (F = 9.88, df = 4, *p* < 0.0001). 

### 3.5. Life Table Parameters

Predicted fecundity for the next generation estimated values were significantly higher at 26 °C (28,439.25) and 32 °C (42,293.72). Similarly, the population trend index estimated using fecundity, sex ratio, and survival data of *D. opuntiae* with an initial population of 200 crawlers in the Morris-Watt life table model [25] indicated a potential population increase of 142.20 and 211.47 times at 26 and 32 °C, respectively, in the next generation (Table 5).

### 3.6. Thermal Requirements

The LDT (°C) and SET (°D) for *D. opuntiae* at five constant temperatures (20–40 °C) were determined by the linear model, as indicated by the high coefficients of determination obtained for all developmental stages of the cochineal (all R^2^ > 0.80) (Table 6). The lower developmental thresholds for *D. opuntiae* ranged from 1.38 °C (young female stage) to 22 °C (first-instar nymph stage); for the total pre-adult female and male and adult female and male stages, they were 10.15, 12.21, 10.54, 21.04 °C, respectively (Table 4). The number of degree-days required for the development of each developmental stage ranged from 22.37 °D (first-instar nymph stage) to 769.23 °D (adult female stage), and to complete development from egg to adult female and male, they were 769.23 and 357.14 °D, respectively (Table 6).

## 4. Discussion

Our study of the effects of temperature on the biology, reproduction, and population growth of *D. opuntiae* showed that the total developmental duration of both *D. opuntiae* females and males decreased significantly as temperature increased from 20 to 40 °C. However, there was a significant increase in the life cycle duration of males between 32 and 40 °C. In this context, Flores-Hernandez et al. [34] reported that temperature has significant effects on life cycle duration, sexual intercourse, and survival of several species of the genus Dactylopius. In this study, eggs underwent an average incubation period ranging from 2.31 h at 40 °C to 23.82 h at 20 °C. Sullivan [30] reported that for the other *Dactylopius* spp, eggs hatch within 3–5 h at 26 °C. The average development time of *D. opuntiae* crawlers from egg to the first molt ranged from 4.82 days at 40 °C to 12.33 days at 20 °C, compared to 15, 18, and 35 days for *D. ceylonicus*, *D. austrinus*, and *Dactylopius tomentosus* (Lamarck, 1801), respectively, at 26 °C [30,46,47]. The female maturity time of 46.36 at 26 °C reported in this study (Table 1) is closer to the female maturity time of 40–50 days for *D. austrinus* at 25 and 26 °C [31,46], *D. opuntiae* at 26 °C [34] and *Dactylopius Coccus* (Costa, 1835) [48] at 26 °C. The average development time of males from egg to death ranged from 26.97 days at 32 °C to 50.75 at 20 °C. These results are in agreement with those found by Flores-Hernández et al. [48]. Furthermore, independent of temperature, our results showed overlaps between stages during the immature period, indicating a variable developmental rate depending on the developmental stage. These results follow the suggestion that the proportion of developmental time of pre-imaginal stages of scale pests is not temperature-dependent but is typical of each stage [49,50].

The most favorable temperature range for survival, development, and reproduction of *D. opuntiae* was found to be 26 to 32 °C. The highest pre-adult accumulated survival rates were recorded at 26 (88%) and 32 °C (93%), and the lowest at 40 (28%) and 20 °C (65%). Moreover, the probability that a newly laid egg would survive to the adult stage was highest at 26 and 32 °C (44–60%) while it was the lowest at 40 °C (6–22%). Furthermore, the first instar crawlers were the most susceptible, and the adult stage was the most resistant to temperature extremes. A comparatively high percentage of adult stage survival has also been reported for *D. austrinus* [31] and *D. ceylonicus* [30]. Hosking [31] and Sullivan [30] had reported that the optimal conditions for growth and development of *D. opuntiae* are 26 °C and 60 ± 5% RH, respectively. Additionally, more females were produced at 40 °C (Table 2), perhaps as a physiological response to the unfavorable high temperatures [25]. This phenomenon has a compensatory effect of increasing the chances for offspring production in the high-temperature region.

The presence of parthenogenesis in females was not observed in our study. The lack of parthenogenesis for other species was also reported for *D. austrinus* [46], *D. ceylonicus* [30], and *D. tomentosus* [47]. The mandatory requirement of males for mating and production of eggs and offspring implies that traps with pheromones could lead to an attraction and destruction strategy for *D. opuntiae*, as suggested for similar amphimictic scale pests [51]. 

The longest oviposition period was observed when the cochineal was reared at 32 °C (17.97), and the shortest was observed at 20 °C (4.72 days), while Luna et al. [52] reported that the oviposition duration of this species lasted six or nine weeks depending on the availability of food at 25 ± 1 °C. Furthermore, the fecundity of *D. opuntiae* was significantly higher at 32 (355.29 egg/female) and 26 °C (325.02 egg/female) and lower at 20 (78.37 egg/female) and 40 °C (55.89 egg/female), the same trend was observed for the percentage of egg hatching since it was significantly higher at 32 (97.75%) and 26 °C (96.92%), and lower at 20 °C (61.55%). The cochineal fecundity in our study was higher than in Flores-Hernandez et al. [34] (131 individuals per female) at 26 °C and lower than in Luna et al. [52] (567.58 ± 164.67 with food (cladodes), and 351.25 ± 131.98 without food) at 25 ± 1 °C. These differences may be due to the host plant used, or to other factors such as the quality of nutrition and stress of the cladodes before removing the females [52]. 

The curves for age-stage specific fecundity (f_x_, female), age-specific fertility (m_x_), and age-specific maternity (l_x_m_x_) showed the highest peaks at 26 and 32 °C. Moreover, the daily fecundity raw data of individual females revealed occasional interruption by days without egg-laying. This may be due to the periodicity of reproductive physiology [53]. Similar results were observed in other scale pest species [53,54].

The total pre-oviposition period, adult pre-oviposition period, and post-oviposition period of *D. opuntiae* at 20 °C were significantly longer than at other temperatures. A possible explanation for this may be linked to the thermal requirements for egg production and maturation in *D. opuntiae*, suggesting that cochineal reproduction may be adversely affected at temperatures below 20 °C [27].

The highest and lowest intrinsic rate of natural increase (r_m_) and the finite rate of increase (λ) were recorded at 32 and 20 °C, respectively. The net reproductive rate (R_0_) was highest at 32 °C, followed by 26 °C, and was much lower at 40 °C. For mean generation time (T) and doubling time values (DT) were higher at 20 °C and lower at 32 °C. At lower (20 °C) and higher (40 °C) temperatures, females produced all eggs and nymphs in a shorter time and then died, while favorable conditions at 26 °C and 32 °C favored the net reproductive rate of females. The parameter (r_m_) is a good indicator for predicting the favorable temperature at which population growth is optimal, as it reflects the overall effect of temperature on survival and biological and reproductive parameters of a population [54]. 

The population trend index indicates that the population growth at the beginning of the next generation would be 142.20 and 211.47 times at 26 and 32 °C, respectively (Table 5). A rapid increase in the population of *D. opuntiae* has implications for its biological control through the use of its natural enemies. Consequently, it is important to note that biological control based solely on the use of the strongest agents is not necessarily capable of limiting populations of this cochineal in the long term [55].

The development rate of *D. opuntiae* females and males varied linearly between 20 and 40 °C (Table 6). The estimated lower development threshold (Tmin) for first instar nymph to adult development in females was 10.15 °C. The lower minimum development threshold of young *D. opuntiae* females (1.38 °C) may explain the greater and more widespread distribution of *D. opuntiae* on cactus crops during summer seasons, as observed by El Aalaoui et al. [12].

In our study, *D. opuntiae* females required a higher thermal constant (769.23 DD) than males (357.14 DD) in order to complete their development and reach the mature (stage of reproduction) adult stage. The population dynamics of *D. opuntiae* in Morocco can be better understood and predicted based on the LDT and SET obtained in this study. In addition, developmental and reproductive data can be used as initial parameters to estimate both the potential spread and relative abundance of *D. opuntiae* [56], which may be useful in developing a better management strategy against this scale pest. 

## 5. Conclusions

Our study generated a wealth of information on the effect of temperature on the survival and biological and reproductive parameters of *D. opuntiae* reared on *O*. *ficus-indica*; this is crucial data for understanding its population dynamics on the cactus. The lower development threshold (LDT) and the sum of effective temperatures (SET) were estimated, and evidence of obligate sexual reproduction was provided against previous erroneous reports that all cochineal insects are parthenogenetic. However, more information regarding the bio-ecology of the cochineal under field conditions is needed to better understand the ecology, dynamics, and population fluctuation of the species, taking into account its hosts, as host nutrition may also influence the survival, life cycle, fecundity, as well as the incidence and severity of the cochineal.

## Figures and Tables

**Table 1:** Developmental duration (mean ± SE) of *D. opuntiae* female and male under different constant temperatures (20, 23, 26, 32, and 40 °C).

Developmental Duration		Temperature ( °C)				
		20	23	26	32	40
Egg incubation period (hours)		23.82 ±0.46 A	21.63 ±0.92 B	17.39 ±0.94 C	7.81 ±0.49 D	2.31 ±0.47 E
First-instar nymph (days)	Female	12.14 ±0.43 A * b **	11.83 ±0.62 Ba	11.03 ±0.45 Ca	2.17 ±0.42 Eb	4.82 ±0.39 Da
	Male	12.33 ±0.65 Aa	11.91 ±0.68 Ba	11.15 ±0.53 Ca	2.36 ±0.57 Ea	5.05 ±0.69 Da
Second-instar nymph (days)	Female	35.20 ±0.51 Aa	23.13 ±0.37 Ba	15.12 ±0.36 Ca	14.20 ±0.50 Da	13.14 ±0.35 Ea
	Male	16.03 ±0.35 Ab	8.14 ±0.35 Cb	7.20 ±0.43 Db	6.18 ±0.42 Eb	13.36 ±0.50 Ba
Third-instar nymph (days)	Young female	25.34 ±0.95 Aa	22.11 ±0.35 Ba	19.19 ±0.48 Da	19.89 ±0.43 Ca	16.16 ±0.37 Ea
	Male Pupal duration (Pupa + Cocoon)	18.21 ±0.56 Ab	14.15 ±0.36 Bb	7.14 ±0.41 Db	9.67 ±0.56 Cb	9.73 ±0.47 Cb
Total pre-adult (days)	Female	73.67 ±1.23 Aa	58.13 ±0.85 Ba	46.36 ±0.75 Ca	37.26 ±0.78 Da	35.11 ±0.69 Ea
	Male	47.10 ±1.90 Ab	35.21 ±0.86 Bb	26.48 ±0.73 Db	19.19 ±0.80 Eb	29.18 ±0.98 Cb
Longevity (days)	Female	45.85 ±1.40 Aa	37.15 ±0.63 Da	43.01 ±0.69 Ca	44.68 ±0.85 Ba	24.59 ±0.73 Ea
	Male	3.32 ±0.73 Cb	5.16 ±0.37 Bb	7.85 ±0.39 Ab	7.78 ±0.42 Ab	2.73 ±0.47 Db
Life cycle (days)	Female	94.23 ±1.75 Aa	73.16 ±1.04 Ba	70.18 ±0.88 Ca	62.05 ±1.04 Da	43.55 ±0.90 Ea
	Male	50.75 ±0.93 Ab	40.35 ±0.86 Bb	34.33 ±0.83 Cb	26.97 ±0.92 Eb	31.91 ±1.14 Db

* Within lines means followed by the same capital letters are not statistically different according to Tukey’s LSD test at α = 0.05; ** For bring up each life stage, within columns means followed by the same lower-case letters are not statistically different according to Fisher’s LSD test, *p* ≤ 0.05.

**Table 2:** Sex ratio (proportion of females) and nymphs mortality rates of *D. opuntiae* population under different constant temperatures (20, 23, 26, 32, and 40 °C).

Temperature (°C)	*n*	Sex Ratio **	The Pre-Adult Survival Rate *
20	129	0.47	0.65
23	164	0.59	0.82
26	175	0.50	0.88
32	186	0.64	0.93
40	55	0.80	0.28

** calculated using all individuals (male and female) in the population; * calculated using all the immature stages in the population.

**Table 3:** Pre-oviposition, oviposition, post-oviposition, fecundity, daily reproduction (eggs/female) and Hatchability (%) of *D. opuntiae* under different constant temperatures (20, 23, 26, 32, and 40 °C) (mean ± SE).

Biological Parameters	Temperature (°C)				
	20 (*n* = 60)	23 (*n* = 96)	26 (*n* = 88)	32 (*n* = 119)	40 (*n* = 44)
Total pre-oviposition period (days)	73.67 ± 1.23 a *	58.13 ± 0.85 b	46.36 ± 0.75 c	37.26 ± 0.78 d	35.11 ± 0.69 e
Adult preoviposition period (days)	25.34 ± 0.95 a	22.11 ± 0.35 b	19.19 ± 0.48 d	19.89 ± 0.43 c	16.16 ± 0.37 e
Oviposition period (days)	4.72 ± 0.69 e	8.55 ± 0.52 c	16.82 ± 0.44 b	17.97 ± 0.37 a	6.20 ± 0.41 d
Post-oviposition period (days)	15.30 ± 0.82 a	5.90 ± 0.34 b	5.93 ± 0.33 b	5.82 ± 0.61 b	3.18 ± 0.39 c
Fecundity	78.37 ± 5.14 d	118.20 ± 3.38 c	325.02 ± 6.46 b	355.29 ± 10.51 a	55.89 ± 2.50 e
Daily reproduction (eggs/female)	16.85 ± 1.83 c	13.86 ± 0.65 d	19.34 ± 0.59 b	19.78 ± 0.64 a	9.03 ± 0.48 e
Maximum daily fecundity	20.5	15	21.31	21.65	10
Hatchability (%)	61.55 ± 2.59 e	78.83 ± 0.60 d	96.92 ± 0.06 b	97.75 ± 0.07 a	91.04 ± 0.41 c

* Means in lines followed by the same lower-case letters are not statistically different according to the Tukey’s LSD test at α = 0.05.

**Table 4:** Intrinsic rate of increase (r), finite rate of increase (λ), net reproductive rate (R_0_), mean generation time (T), and doubling time (DT) of *D. opuntiae* under different constant temperatures (20, 23, 26, 32, and 40 °C) (mean ± SE).

Population Growth Parameters	Temperature (°C)				
	20	23	26	32	40
r	0.041 ± 0.001 e *	0.065 ± 0.000 d	0.092 ± 0.000 b	0.119 ± 0.000 a	0.066 ± 0.000 c
λ	1.042 ± 0.001 e	1.067 ± 0.000 d	1.096 ± 0.000 b	1.126 ± 0.000 a	1.068 ± 0.000 c
R_0_	23.47 ± 0.29 d	56.73 ± 0.03 c	143.00 ± 0.07 b	211.36 ± 0.21 a	12.30 ± 0.00 e
T	76.47 ± 16.93 a	62.36 ± 10.88 ab	54.26 ± 6.93 ab	44.43 ± 6.60 b	38.75 ± 22.80 b
DT	16.79 ± 0.34 a	10.74 ± 0.00 b	7.55 ± 0.00 d	5.82 ± 0.00 e	10.55 ± 0.00 c

* Means in lines followed by the same lower-case letters are not statistically different according to the Tukey’s LSD test at α = 0.05.

**Table 5:** Life table parameters of *D. opuntiae* at different constant temperatures (20, 23, 26, 32, and 40 °C).

Life Table Parameters	Temperature (°C)				
	20	23	26	32	40
Number of initial crawlers (N_0_)	200	200	200	200	200
Number of first-instar nymph female	70	100	94	121	55
Number of first-instar nymph male	90	75	94	70	20
Number of second-instar nymph female	65	98	90	119	49
Number of second-instar nymph male	80	70	89	67	14
Number developing into young female	61	96	88	119	44
Number developing into cocoon	72	68	87	67	11
Number developing into adults (A)	133	164	175	186	55
Number developing into mature female	61	96	88	119	44
Number developing into adult male	72	68	87	67	11
Predicted fecundity of next generation (G_2_ = A × P_♀_ × P_F_)	4898.91	11,437.03	28,439.25	42,293.72	2459.16
Population trend index I = G2/N0	24.49	57.18	142.20	211.47	12.30

P_♀_ = Female proportion, P_F_ = Average fecundity per female.

**Table 6:** Lower development threshold (LDT) and sum of effective temperatures (SET) required for the development of the different stages of *D. opuntiae*, with the respective coefficient of determination (R^2^), slope (a), and intercept (b).

Life Stages	1/DT=aT+b		R^2^	LDT (°C)	SET (°D)
	a	b			
First-instar nymph female	0.0447	−0.9936	0.90	22.23	22.37
First-instar nymph male	0.0403	−0.8893	0.90	22.07	24.81
Second-instar nymph female	0.0063	−0.0987	0.98	15.67	158.73
Second-instar nymph male	0.0128	−0.1852	0.90	14.47	78.13
Young female	0.0021	−0.0029	0.99	1.38	476.19
Cocoon	0.0142	−0.2378	0.90	16.75	70.42
Total pre-adult female	0.0013	−0.0132	0.99	10.15	769.23
Total pre-adult male	0.0028	−0.0342	0.99	12.21	357.14
Adult female	0.0013	−0.0137	0.80	10.54	769.23
Adult male	0.0177	−0.3724	0.82	21.04	56.50

## Data Availability

Data sharing not applicable.
